# Disseminated tuberculosis presented with explicit hypercalcemia: A clinical case report

**DOI:** 10.1002/ccr3.8507

**Published:** 2024-02-09

**Authors:** Seyed Ali Dehghan Manshadi, Nahid Shafiee, Seyed Mohammad Piri, Erfan Naghavi, Maryam Moradi

**Affiliations:** ^1^ Department of Infectious Disease and Tropical Medicine/ Iranian Research Center for HIV/AIDS (IRCHA) Tehran University of Medical Sciences Tehran Iran; ^2^ Department of Infectious Disease and Tropical Medicine Tehran University of Medical Sciences Tehran Iran; ^3^ Cancer Research Institute Department of Pathology Tehran University of Medical Sciences Tehran Iran; ^4^ School of Medicine Tehran University of Medical Sciences Tehran Iran; ^5^ Eye Research Center, The Five Senses Health Institute, Rassoul Akram Hospital Iran University of Medical Sciences Tehran Iran

**Keywords:** hypercalcemia, TB‐related hypercalcemia, tuberculosis (TB)

## Abstract

**Key Clinical Message:**

Tuberculosis (TB) is a rare but known reason for hypercalcemia usually in those with underlying conditions such as renal failure, diabetes, or severe anemia. It is essential to consider TB in those with refractory or resistant hypercalcemia.

**Abstract:**

Hypercalcemia or a calcium level above 10.5 mg/dL can be a manifestation of TB that only became symptomatic in a small percentage of the patients. Patients with underlying diseases such as renal failure are more prone to poor prognosis. It is essential to use anti‐TB drugs besides hypercalcemia standard treatment to maintain a normal calcium level in TB‐related hypercalcemia. In thisstudy, we have presented a young adult with disseminated TB and persistent hypercalcemia who responded finally to anti‐TB drugs.

## 1 INTRODUCTION

Tuberculosis (TB) is an ancient multi‐systemic disease caused by mycobacterium tuberculosis. Its manifestations are usually those regarding pulmonary involvement, such as chronic cough, fever, weight loss, and night sweats, while it can present with various symptoms including hypercalcemia. According to the WHO report, the incidence rate has increased by 3.6% in 2021 affecting more than 10 million people worldwide.^1^, ^2^

Calcium is an important cation functioning in numerous critical processes in the human body. Musculoskeletal, nervous, and hematological systems are fundamental apparatuses in which calcium gets involved.^3^ Hypercalcemia is defined by the high amount of calcium in the blood, more than what is considered normal in a laboratory test (usually greater than 10.5 mg/dL). Most laboratory analyses report the total amount of calcium in the blood, even though only a free ionized (45%) form of calcium is used by cells and can activate important processes.^4^

Malignancies are the most common cause of hypercalcemia, especially in those with bone metastasis. Other causes include severe dehydration, immobility, hyperparathyroidism, genetic disorders (familial hypocalciuric hypercalcemia), certain medications (lithium) and supplements, and also some granulomatous diseases such as TB and sarcoidosis.^5^ It has been reported that hypercalcemia was presented in one‐fifth of the patients diagnosed with TB.^6^

Standard treatment for hypercalcemia is intravenous fluids, calcitonin, and intravenous bisphosphonates. Resistant hypercalcemia outcrops when blood calcium levels continue to be high even after trying different treatments, or if therapeutic interventions need to be repeated within 2 weeks of the first treatment.^7^, ^8^

In some rare cases of TB, severe hypercalcemia can be detected. Excessive extra‐renal 1‐alpha hydroxylase activity, elevated creatinine level, renal failure, diuretics consumption, diabetes mellitus, severe anemia, and disseminated TB are explained as TB‐related hypercalcemia reasons.^6^, ^9^ In this study, we are presenting a patient with persistent hypercalcemia due to disseminated TB.

## 2 CASE PRESENTATION

A 28‐year‐old nonsmoking Afghan male was admitted to the infectious diseases ward of Imam‐Khomeini Hospital Complex (IKHC), Tehran, Iran in March 2023, with severe abdominal pain, fever, night sweats, and significant weight loss of 15 pounds within 1 month without any underlying disease. He reported colic abdominal pain in the epigastric and paraumbilical regions about 20 days before admission. The pain worsened with the ingestion of food and was associated with nausea and nonbloody vomiting. He experienced constipation and anorexia along with urinary frequency when he was admitted to the hospital. The patient did not reported any respiratory problems or any history of pulmonary diseases.

At the time admission, he was pale and had a blood pressure of 120/80 mmHg, pulse rate of 110/min, respiratory rate of 29/min, and temperature of 39°C. On abdominal examination, obvious distention with guarding along with severe tenderness in the epigastric area was detected.

Primary and advanced laboratory analysis, abdominal and chest computed tomography (CT) scan, and surgical consult were done. Laboratory test results are summarized in Table 1.

**TABLE 1:** Laboratory test results.

WBC	10.4 × 10^9^/L (4.5–11.0 × 10^9^/L)^a^	Sodium	135 mEq/L (135–145)
RBC	3.55 million cells/mcL (4.35–5.65)	Potassium	3.3 mmol/L (3.6–5.2)
Hb	8.2 g/dL (14–18)	Magnesium	1.8 mg/dL (1.7–2.2)
HCT	28.5% (40%–54%)	Urea	51 mg/dL (6–24)
MCV	80.3 fL (80–100)	Creatinine	1.4 mg/dL (0.7–1.3)
MCH	23.1 pg/cell (27–31)	Calcium	12.9 mg/dL (8.6–10.3)
MCHC	28.8 g/dL (32–36)	Albumin	2.5 g/dL (3.4–5.4)
PLT	584,000/μL (150,000–450,000)	Corrected Ca Level	14.1 mg/dL (8.5–10.2)
RDW	49% (12%–15%)	PTH	<0.3 (14–65 pg/mL)
PT	15.1 s (10–13)	Total protein	6.3 g/dL (6.0–8.3)
INR	1.33 (2.0–3.0)	Phosphor	3.6 mg/dL (2.5–4.5)
PTT	30 s (25–35)	ANA	Negative
FBS	103 mg/dL (70–100)	Anti‐ds‐DNA	Negative
AST	18 U/L (14–20)	CH50	118 (70–150 U/mL)
ALT	20 U/L (29–33)	P‐ANCA	Negative
ALK.P	379 IU/L (44–147)	C‐ANCA	Negative
LDH	228 U/L (140–280)	U/A, U/C, B/C	Negative
Bill. T	0.4 mg/dL (less than 1)	Pro‐calcitonin	1.73 (Normal<0.3 ng/mL)
Bill. D	0.2 mg/dL (less than 0.3)	GBM Ab	1.42 (positive ≥3 U/mL)
25 OH Vit D3	28.26 ng/mL (30–50)	HIV	Negative

^a^ Normal ranges are provided within the parenthesis.

Abbreviations: ALK.P, alkaline phosphatase; ALT, alanine transaminase; ANA, antinuclear antibody; Anti‐ds‐DNA, anti‐double‐stranded deoxyribonucleic acid antibodies; AST, aspartate transaminase; B/C, blood culture; Bill.D, direct billirobinm; Bill.T, tolal billirobin; C‐ANCA, antineutrophil cytoplasmic autoantibody; CH50, complement total activity; FBS, fasting blood suger; GBM Ab, anti–glomerular basement membrane antibody; Hb, hemoglobulin; HCT, hematocrite; INR, international normalized ratio; LDH, lactate dehydrogenase; MCH, mean corpuscular hemoglobin; MCHC, mean corpuscular hemoglobin concentration; MCV, mean corpuscular volume; P‐ANCA, perinuclear anti‐neutrophil cytoplasmic antibodies; PLT, platelet; PT, prothrombin time; PTH, parathyroid hormone; PTT, partial tromboplastin time; RBC, red blood cell; RDW, red cell distribution width; U/A, urine analysis; U/C, urine culture; WBC, white blood cell.

Sputum smear for Acid‐Fast bacilli was negative 3 times. The sputum specimen was sent for culture and PCR which were both positive for TB after 72 h.

**FIGURE 1:**
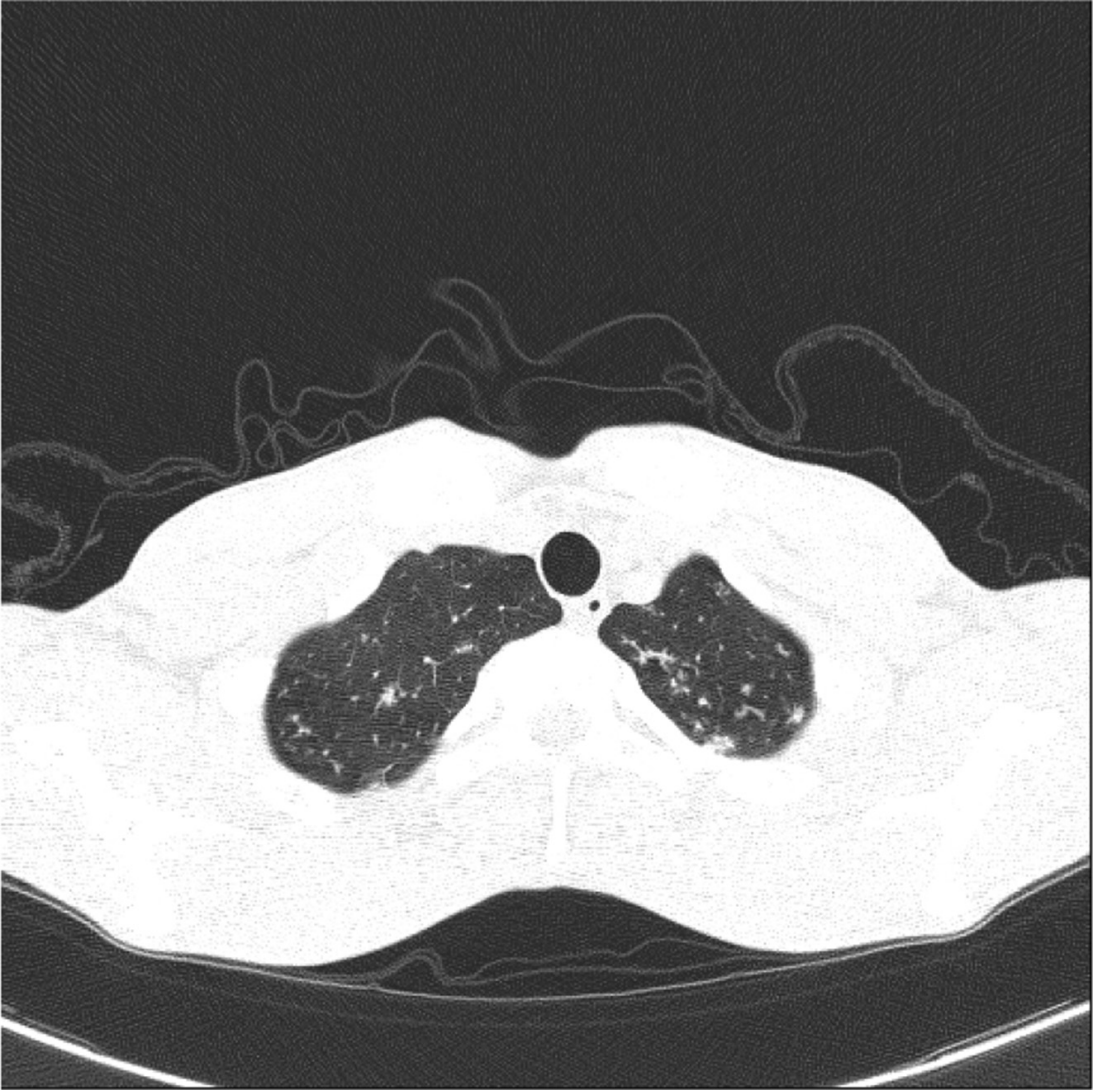
Chest CT scan. Tree in bud pattern in superior lobes.

**FIGURE 2:**
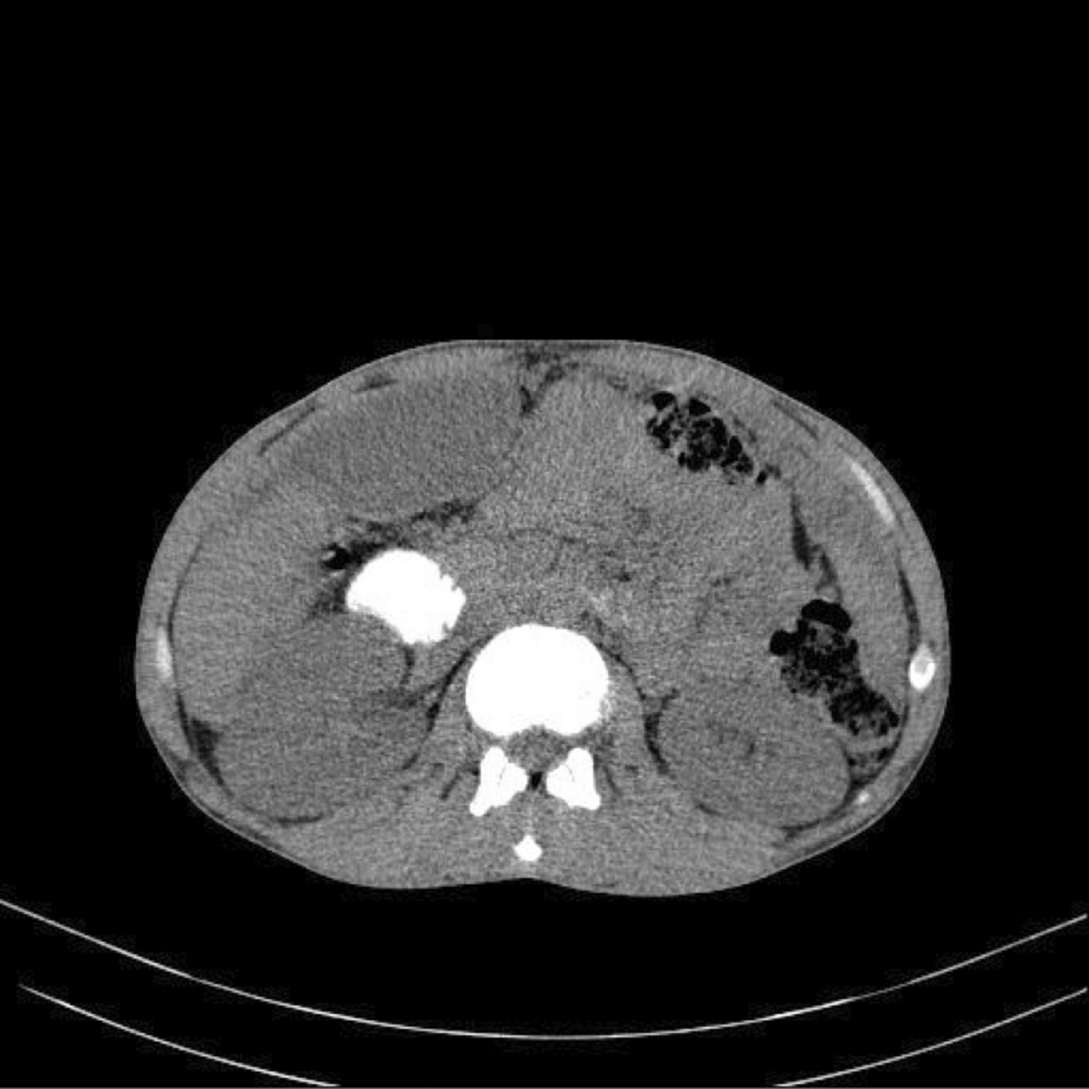
Abdominal CT scan. Increased in the omentum, nodularity, and a subdiaphragmatic abscess (66*148 mm).

**FIGURE 3:**
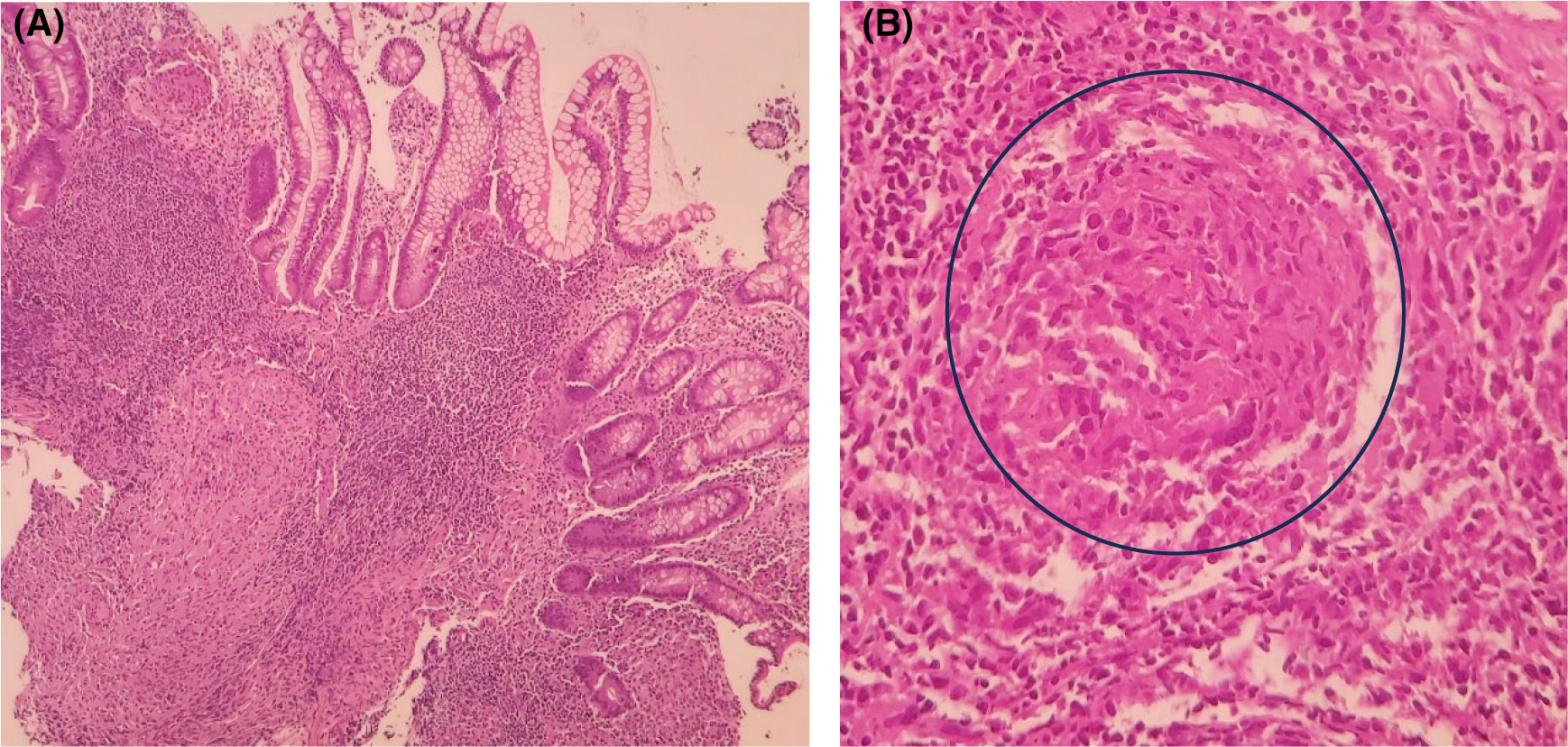
Pathological slides. (A) Microscopic examination (×10) shows terminal ileal mocusa with preserved architecture and some well‐formed granulomas in deep lamina propria favoring granulomatous pattern. (B) High magnification (×40) of granulomas composed of giant cells (shown in circle) and epithelioid histiocytes favoring granulomatous pattern.

On the chest CT scan (Figure 1), a suspicious tree‐in‐bud view in the superior lobes was seen. Increased in the omentum, nodularity, and a subdiaphragmatic abscess (66*148 mm) were detected on the abdominal CT scan (Figure 2). The abscess was drained and the result was positive for Acid‐Fast bacilli and MTB‐PCR. A colonoscopy was done due to the weight loss, anemia, and abdominal pain. The ileocecal valve was edematous and ulcerated. The terminal ileum was edematous and hyperemic, with a cobble‐stoning appearance suggestive of Crohn's or TB. Biopsy was taken from ulcers. Granulomas composed of giant cells and epithelioid histiocytes were seen, which were in favor of TB (Figure 3).

Based on the clinical manifestations, laboratory test results and paraclinical imaging, TB was diagnosed. A 4‐drug regimen of isoniazid, rifampin, pyrazinamide, and ethambutol was initiated. Treatment of hypercalcemia started promptly after admission before completion of patients' evaluation with intravenous fluids, calcitonin, and intravenous bisphosphonates, but improvement in blood calcium level did not occur until the administration of anti‐TB drugs when hypercalcemia corrected within a few days of TB chemotherapy. See also Table 2 for detailed measured calcium levels during hospitalization.

**TABLE 2:** Corrected calcium level changes during hospitalization.

Day of hospitalization	Calcium level
At admission	14.1 mg/dL
Day 1	13.8 mg/dL
Day 2	13.7 mg/dL
Day 3	13.6 mg/dL
Day 3 (After initiation of the 4‐drug regimen)	13 mg/dL
At discharge	11 mg/dL

The patient was discharged with an acceptable clinical appearance with Isoniazid 300 mg, rifampin 600 mg, pyrazinamide 1500 mg, and ethambutol 800 mg daily for 1 year due to the uncommon complication and Vitamin D 1000 mg, vitamin B6, and calcium supplements daily for a month. After 3 months, the patient presented with weight gain and good clinical condition, and anti‐TB therapy was going on without any side effects. Due to the clinical improvement and normal calcium levels in the follow‐up session, the patient was not given any corticosteroids.

## 3 DISCUSSION

Hypercalcemia has been demonstrated in patients with granulomatous disorders that cause inflammation such as sarcoidosis and TB; however, only a small percentage of these patients are symptomatic. About 2.3%–28% of the patients with TB, depending on the group of people being studied in different countries, manifest hypercalcemia. Excess 1, 25‐dihydroxy vitamin D3 levels from 25‐dihydroxy vitamin D3 conversion (vitamin D dysregulation) are described as a diagnostic factor in most patients with TB‐related hypercalcemia.^10^ Hypercalcemia in granulomatous disorders can be difficult to diagnose. Keeping strong suspicion and being aware of the symptoms can help early detection and treatment.^11^

TB‐related hypercalcemia can occur whenever the infection is active. Hypercalcemia can be an initial manifestation of tuberculous peritonitis if there are no other signs and symptoms. This condition is worse in those on routine dialysis, elderly patients, and those with concomitant diseases. It is important to consider TB, especially in endemic areas in those with atypical or resistant hypercalcemia.^12^

There have been reports on a patient presented with pulmonary TB and delirium secondary to a hypercalcemic crisis which was eventually resolved with anti‐TB treatments along with palliative care.^13^

In patients with TB‐related hypercalcemia, standard treatment along with corticosteroids and anti‐TB regimens are needed to resolve the hypercalcemia.^14^ Our patient did not respond to the standard treatment alone but after the 4‐drug regimen initiation, his hypercalcemia was corrected even without steroid courses. Excellent clinical response to prednisone was reported in a case of refractory TB adenopathy with hypercalcemia even without any response to anti‐TB treatments.^15^

This case highlights a young adult without any prior medical history, with disseminated TB and TB‐related hypercalcemia which will respond to anti‐TB treatment. We want to emphasize how important it is to suspect granulomatous‐induced hypercalcemia, even though it is not very common.

## 4 CONCLUSION

TB is a rare but known reason of hypercalcemia usually in those with underlying conditions such as renal failure, diabetes, or severe anemia. It is essential to consider TB in those with refractory or resistant hypercalcemia even without any underlying diseases.

## AUTHOR CONTRIBUTIONS

**Seyed Ali Dehghan Manshadi:** Conceptualization. **Nahid Shafiee:** Project administration. **Seyed Mohammad Piri:** Resources. **Erfan Naghavi:** Resources. **Maryam Moradi:** Conceptualization; writing – original draft.

## CONFLICT OF INTEREST STATEMENT

The authors declare that they have no competing interests.

## CONSENT

Written informed consent was obtained from the patient to publish this report in accordance with the journal's patient consent policy.

## Data Availability

Data sharing does not apply to this article as no datasets were generated or analyzed during the current study.
